# Impact of the weekend effect on outcome after microsurgical clipping of ruptured intracranial aneurysms

**DOI:** 10.1007/s00701-020-04689-9

**Published:** 2021-01-05

**Authors:** Lukas Goertz, Christoph Kabbasch, Muriel Pflaeging, Lenhard Pennig, Kai Roman Laukamp, Marco Timmer, Hanna Styczen, Gerrit Brinker, Roland Goldbrunner, Boris Krischek

**Affiliations:** 1 Center for Neurosurgery, Medical Faculty and University Hospital, University of Cologne, Kerpener Strasse 62, 50937 Cologne, Germany; 2 Department of Neuroradiology, Medical Faculty and University Hospital, University of Cologne, Kerpener Strasse 62, 50937 Cologne, Germany; 3 Institute for Diagnostic and Interventional Radiology and Neuroradiology, University Hospital Essen, Hufelandstraße 55, 45147 Essen, Germany; 4 Department of Neurosurgery , Hôpitaux Robert Schuman , 9 Rue Edward Steichen, 2540 Luxembourg, Luxembourg

**Keywords:** Angiographic outcome, Cerebral infarction, Clipping, Modified Rankin scale, Functional outcome, Night

## Abstract

**Background:**

The “weekend effect” describes the assumption that weekend and/or on-call duty admission of emergency patients is associated with increased morbidity and mortality rates. For aneurysmal subarachnoid hemorrhage, we investigated, whether presentation out of regular working hours and microsurgical clipping at nighttime correlates with worse patient outcome.

**Methods:**

This is a retrospective review of consecutive patients that underwent microsurgical clipping of an acutely ruptured aneurysm at our institution between 2010 and 2019. Patients admitted during (1) regular working hours (Monday–Friday, 08:00–17:59) and (2) on-call duty and microsurgical clipping performed during (a) daytime (Monday–Sunday, 08:00–17:59) and (b) nighttime were compared regarding the following outcome parameters: operation time, treatment-related complications, vasospasm, functional outcome, and angiographic results.

**Results:**

Among 157 enrolled patients, 104 patients (66.2%) were admitted during on-call duty and 48 operations (30.6%) were performed at nighttime. Admission out of regular hours did not affect cerebral infarction (*p* = 0.545), mortality (*p* = 0.343), functional outcome (*p* = 0.178), and aneurysm occlusion (*p* = 0.689). Microsurgical clipping at nighttime carried higher odds of unfavorable outcome at discharge (OR: 2.3, 95%CI: 1.0–5.1, *p* = 0.039); however, there were no significant differences regarding the remaining outcome parameters. After multivariable adjustment, clipping at nighttime did not remain as independent prognosticator of short-term outcome (OR: 2.1, 95%CI: 0.7–6.2, *p* = 0.169).

**Conclusions:**

Admission out of regular working hours and clipping at nighttime were not independently associated with poor outcome. The adherence to standardized treatment protocols might mitigate the “weekend effect.”

## Introduction

Aneurysmal subarachnoid hemorrhage (aSAH) is a severe neurological condition caused by spontaneous rupture of intracranial aneurysms. In order to reduce the risk of aneurysm rebleeding and further potential brain damage, timely aneurysm occlusion is advocated [17].

Microsurgical clipping represents a well-established, safe and effective technique for aneurysm occlusion, in particular for complex aneurysms, which are challenging to treat by endovascular means [11–13]. When patients are admitted during the weekend or during nighttime, the question arises, whether microsurgical clipping should be performed during on-call duty or postponed to the next working day. Previous studies suggested that emergency hospital admissions and/or procedures performed at night and on weekends may be associated with increased morbidity and mortality when compared to admissions during the routine daytime shift [3]. This phenomenon was denoted as the “weekend effect” [20]. Although there is some controversy on this subject, this effect was reported for several neurological diseases, such as ischemic stroke [18], intracranial hemorrhage [9], and subarachnoid hemorrhage [27]. The cause of this effect has not yet been determined with absolute certainty. Possible reasons are, for example, a reduction of both the medical staff and availability of resources and organizing factors outside routine working times [20]. Moreover, due to the human circadian rhythm, cognitive performance varies throughout the day and usually reaches its lowest point at night, possibly yielding reduced overall quality of patient management [29]. For aSAH patients, the association between timing of microsurgical clipping, surgical performance and clinical outcome has not yet been analyzed systematically. The objective of this study was to evaluate, whether admission of patients with aSAH out of regular working hours and/or microsurgical clipping performed at night is associated with a worse patient outcome. For this purpose, the following outcome parameters were defined: operation time, cerebral infarction, in-hospital mortality, functional outcome and angiographic results.

## Methods

Consecutive aSAH patients that underwent microsurgical clipping of the index aneurysm at the University hospital of Cologne between January 2010 and December 2019 were retrospectively reviewed. The catchment area consists around 700,000 inhabitants. Aneurysms are treated by 3 to 4 vascular neurosurgeons per year that are on call alternately. At the author’s institution, around 140 aneurysms (70 ruptured, 70 unruptured) are treated per year, thereof 40 by clipping and 100 by endovascular means. There are no hybrid vascular neurosurgeons. Endovascular aneurysm treatment is performed by interventional neuroradiologists. The study was approved by the local ethics committee (Registration ID: 13–104) and was conducted in accordance with the Declaration of Helsinki.

### Study enrollment

All patients that underwent microsurgical clipping for a ruptured intracranial aneurysm within 14 days after ictus were considered for study enrollment. Specific exclusion criteria were the following: (1) recurrent aneurysms, (2) dissecting aneurysms, (3) fusiform aneurysms, (4) mycotic aneurysms, (5) blister aneurysms, (6) giant aneurysms (diameter > 25 mm), and (7) previously treated aneurysms.

### Procedural details

Upon radiological proof of aSAH, either computed tomography angiography (CTA) and/or digital subtraction angiography (DSA) were performed in order to determine aneurysm location, size, morphology and vascular anatomy. The institutional treatment protocol consists of treating ruptured intracranial aneurysms as soon as possible after diagnosis, both for patients with intracranial hemorrhage and without. Ultimately, the operation starts at the discretion of the vascular neurosurgeon on duty, which depends on several factors such as case volume, clinical patient condition and time of the night. Microsurgical clipping was performed using an OPMI® PENTERO® 800 operation microscope with integrated FLOW 800 module (Carl Zeiss AG, Oberkochen, Germany). Aneurysm occlusion and parent artery patency was intraoperatively evaluated by the use of micro-Doppler ultrasound and/or indocyanine green (ICG)-videoangiography (VAG) with additional FLOW 800 analysis of cerebral perfusion [15, 16]. After surgery, the patients were surveilled at an intensive care at least until 14 days after the initial bleeding. Within 24 h after surgery, the patients received a cranial computed tomography scan to exclude rebleeding and treatment-related cerebral infarction. Transcranial Doppler ultrasound was performed daily, considering a mean cerebral blood flow velocity ≥ 120 cm/s and/or an increase by ≥ 50 cm/s within 24 h as indicative for cerebral vasospasm. In this case, the patients underwent a cranial CT scan with angiography and perfusion for radiological proof of vasospasm.

### Data collection

The following parameters were collected retrospectively from the medical charts: patient age, gender, day and time of admission, World Federation of Neurosurgical Societies (WFNS) grading scale score, Fisher score, and neurological status at discharge and at 6-month follow-up. Operation records were reviewed to retrieve the following procedural specifics: day and time of surgery, admission-to-surgery time (time interval between admission and start of surgery), operation time (interval between skin incision and suture), use of micro-Doppler and/or ICG-VAG, temporary clipping, and intraoperative rupture. Preoperative CTA and DSA scans were reviewed to determine the aneurysm location, size (i.e. largest aneurysm diameter), neck width, morphology (regular/irregular), calcification of the aneurysm wall, partial thrombosis and vessels arising from the aneurysm sac. Following the criteria proposed by Andaluz et al., and in consideration of the exclusion criteria, location at the posterior circulation, a neck width > 6 mm, lobulated morphology, calcification of the aneurysm wall, intrasaccular thrombosis, and vessels arising from the aneurysm sac were defined as complex aneurysms [1, 14]. Treatment-related infarction was defined as any new ischemic lesion on postoperative CT within 48 h after surgery that could be clearly related temporally and spatially to the clipping procedure and the parent artery of the treated aneurysm. The CT and magnetic resonance imaging (MRI) scans were thoroughly reviewed to evaluate if vasospasm was present and may have caused the cerebral ischemia. To evaluate functional outcome, the modified Rankin scale (mRS) score was determined at discharge and at 6-month follow-up. A mRS score ≤ 2 was defined as a favorable outcome and a mRS score of 3-6 as unfavorable outcome. A mRS of 6 indicates death. Selected patients received angiographic control of aneurysm occlusion, either at the end of their hospital stay or at follow-up visits. The Raymond-Roy occlusion classification (RROC) was applied to assess aneurysm occlusion: (1) complete occlusion, (2) neck remnant, and (3) aneurysm remnant.

### Time of treatment

The patient cohort was categorized based on two classification schemes:

1. Based on the day and time of admission, the patients were divided into (1) admission during standard working hours (Monday to Friday, 08:00–17:59), and (2) admission during weekday on-call duty (18:00–07:59), weekends or public holidays.
2. Based on the time of skin incision, the surgery was defined to be performed during (1) daytime (08:00–17:59), and (2) nighttime (18:00–07:59).

### Statistical analysis

Baseline patient and aneurysm characteristics were analyzed using descriptive statistics. To compare categorical variables, the Chi-Square and the Fisher’s exact tests were used, when appropriate. Continuous variables were presented as means ± standard deviation and tested for normality using the Shapiro-Wilk test. Groups were compared using the two-sided unpaired Student’s t test (for normally distributed data) and the Mann-Whitney U test (for non-normally distributed data). Factors predictive for unfavorable functional outcome in the univariate analysis (*p* < 0.1) were entered into a binary logistic stepwise regression model to identify factors independently associated with clinical outcome. All calculations were performed using SPSS software (version 25, IBM SPSS Statistics for Windows, Armonk, NY, USA). A *p* value < 0.05 was considered statistically significant.

## Results

### Patient and aneurysm characteristics

**Fig. 1:**
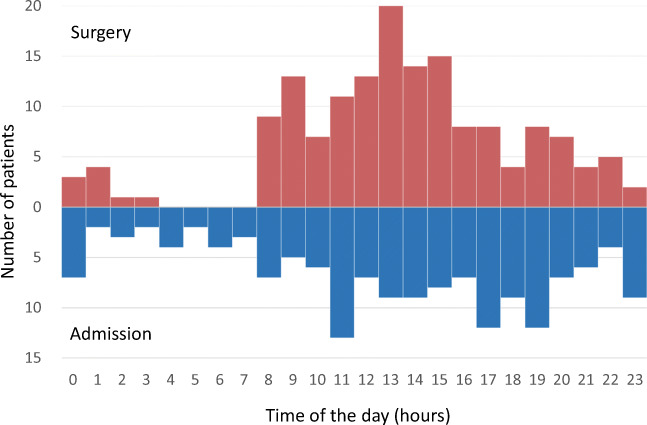
Distribution of patient admission start of surgery in dependence of the time of the day

A total of 157 patients met the inclusion criteria and were enrolled. The distribution of patient admission and start of surgery in dependence of the time of the day is shown in Fig. 1. The mean patient age was 55.4 ± 13.4 years and 113 patients were female (72.0%). Fifty-five patients (35.0%) presented with WFNS grade 4 or 5 and 90 had a Fisher 4 hemorrhage (57.3%). Among 157 ruptured aneurysms, 45 (28.7%) were located at the anterior cerebral artery, 75 (47.8%) at the middle cerebral artery, 31 (19.7%) at the internal cerebral artery and 6 (3.8%) at the posterior circulation. The mean aneurysm size was 7.4 ± 3.4 mm and the mean neck width was 3.8 ± 1.7 mm. Irregular shape was seen in 127 aneurysms (80.9%). Seventeen aneurysms (11.1%) had a neck width > 6 mm, 55 (35.0%) were lobulated, 19 (12.1%) had calcifications of the aneurysm wall, 2 (1.3%) were partially thrombosed, and 9 (5.7%) had vessels arising from the aneurysm sac. Eighty-five aneurysms (54.1%) were defined as complex aneurysms.

Microsurgical clipping was performed by 7 vascular neurosurgeons during the study period. Table 1 shows how many aneurysms were clipped during standard working hours, on-call duty, and nighttime by each individual neurosurgeon. In three patients, coiling of the ruptured aneurysm was attempted but failed and the aneurysms were subsequently treated by microsurgical clipping. The mean admission-to-surgery time was 9.2 ± 6.8 h and the mean operation time was 270 ± 80 min. Micro-Doppler ultrasound was used in 91.7%, ICG-VAG in 85.3% and temporary parent artery clipping was performed in 40.7%. Intraoperative rupture occurred in 26.1% and treatment-related cerebral infarction in 21.7%. Ninety patients developed vasospasm (57.3%) and the overall cerebral infarction rate was 45.2%. The in-hospital mortality rate was 13.4%, while 32.4% achieved a favorable outcome at discharge and 49.0% at follow-up. Among 89 patients with available angiographic follow-up, 73.0% had complete occlusion, 20.3% had neck remnants, and 6.7% had aneurysm remnants.

**Table 1:** Number of aneurysms treated by each individual neurosurgeon during standard working hours, on-call duty and at nighttime

Neurosurgeon	Standard working hours	On-call duty/nighttime	Total aneurysm number
1	19 (47.5%)	21 (52.5%)/13 (32.5%)	40
2	11 (33.3%)	22 (66.7%)/12 (36.4%)	33
3	3 (100%)	0 (0%)/0 (0%)	3
4	8 (26.7%)	22 (73.3%)/12 (40.0%)	30
5	11 (68.8%)	5 (31.2%)/2 (12.5%)	16
6	2 (100%)	0 (0%)/0 (0%)	2
7	19 (57.6%)	14 (42.4%)/9 (27.3%)	33

### Admission during standard working hours vs. on-call duty

Stratifying for admission day and time, 53 patients (33.8%) were admitted during standard working hours and 104 (66.2%) during on-call duty. Thereof, 74 patients (47.1%) were admitted during nighttime. Baseline patient and aneurysm characteristics were comparable between both groups, as detailed in Table 2. For patients admitted during standard working hours, the mean admission-to-surgery time (8.4 ± 7.4 h vs. 9.7 ± 6.4 h, *p* = 0.289) and the mean operation time (282 ± 83 min vs. 264 ± 78 min, *p* = 0.173) were comparable to patients admitted during on-call duty. There were no significant differences regarding procedural specifics and procedural complications, as specified in Table 3. During the hospital stay, a similar portion of patients in both groups developed vasospasm (*p* = 0.833) and cerebral infarction (*p* = 0.491). Favorable outcome was by trend more often achieved by patients which were admitted during on-call duty (37.5% vs. 22.6%, *p* = 0.060), while in-hospital mortality rates were comparable between the groups (17.0% vs. 11.5%, *p* = 0.343). The difference in functional outcome was mitigated at 6-month follow-up (52.9% vs. 41.5%, *p* = 0.178). Complete occlusion rates at angiographic follow-up were similar in both groups (75.9% vs. 71.7%, *p* = 0.689). Patient outcome is described in detail in Table 4.

**Table 2:** Baseline patient and aneurysm characteristics. *WFNS* World Federation of Neurosurgical Societies, *ACA* anterior cerebral artery, *MCA* middle cerebral artery, *ICA* internal cerebral artery, *PC* posterior circulation

Variable	Standard working hours (*N* = 53)	On-call duty (*N* = 104)	*p*	Daytime (*N* = 109)	Nighttime (*N* = 48)	*p*
Age (years)	56.7 ± 11.8	54.7 ± 14.1	0.383	54.3 ± 13.1	57.8 ± 13.9	0.139
Gender			0.420			0.551
Female	36 (67.9%)	77 (74.0%)		80 (73.4%)	33 (22.9%)	
Male	17 (32.1%)	27 (26.0%)		29 (26.6%)	15 (12.5%)	
WFNS grade			0.550			0.481
WFNS 1	18 (34.0%)	28 (26.9%)		35 (32.1%)	11 (27.1%)	
WFNS 2	9 (17.0%)	16 (15.4%)		19 (17.4%)	6 (10.4%)	
WFNS 3	8 (15.1%)	23 (22.1%)		18 (16.5%)	13 (27.1%)	
WFNS 4	3 (5.7%)	12 (11.5%)		10 (9.2%)	5 (10.4%)	
WFNS 5	15 (28.3%)	25 (24.0%)		27 (24.8%)	13 (27.1%)	
Fisher grade			0.494			0.299
Fisher 1	1 (1.9%)	3 (2.9%)		3 (2.8%)	1 (2.1%)	
Fisher 2	7 (13.2%)	7 (6.7%)		9 (8.3%)	5 (10.4%)	
Fisher 3	14 (26.4%)	35 (33.7%)		39 (35.8%)	10 (20.8%)	
Fisher 4	31 (58.5%)	59 (56.7%)		58 (53.2%)	32 (66.7%)	
Aneurysm location						
ACA	18 (34.0%)	27 (26.0%)	0.294	33 (30.3%)	12 (25.0%)	0.501
MCA	24 (45.3%)	51 (49.0%)	0.656	48 (44.0%)	27 (56.3%)	0.158
ICA	10 (18.9%)	21 (20.2%)	0.844	23 (21.1%)	8 (16.7%)	0.520
PC	1 (1.9%)	5 (4.8%)	0.367	5 (4.6%)	1 (2.1%)	0.668
Aneurysm size (mm)	7.4 ± 3.2	7.4 ± 3.5	0.980	7.3 ± 3.3	7.6 ± 3.7	0.513
Neck width (mm)	4.0 ± 1.9	3.8 ± 1.7	0.503	3.9 ± 1.8	3.7 ± 1.5	0.528
Irregular shape	42 (79.2%)	85 (81.7%)	0.708	87 (79.8%)	40 (83.3%)	0.606
Complex aneurysms	29 (54.7%)	56 (53.8%)	0.328	61 (56.0%)	24 (50.0%)	0.490

**Table 3:** Procedural specifics and complications. *ICG-VAG* indocyanine-videoangiography, *n.r.* not reported

Variable	Standard working hours (*N* = 53)	On-call duty (*N* = 104)	*p*	Daytime (*N* = 109)	Nighttime (*N* = 48)	*p*
Admission-to-surgery time (hours)	8.4 ± 7.4	9.7 ± 6.4	0.289	11.1 ± 6.9	5.0 ± 3.9	< 0.001
Operation time (min)	282 ± 83	264 ± 78	0.173	274 ± 84	261 ± 70	0.337
Micro-Doppler	51 (96.2%)	93 (89.4%)	0.144	102 (93.6%)	42 (87.5%)	0.203
ICG-VAG	45 (84.9%)	89 (85.6%)	0.910	98 (89.9%)	36 (75.0%)	0.015
Multiple aneurysm clips (> 1)	14 (26.4%)	35 (33.7%)	0.355	33 (30.3%)	16 (33.3%)	0.703
Temporary clipping						
Yes	18 (34.0%)	37 (35.6%)	0.978	39 (35.8%)	16 (33.3%)	0.966
No	26 (49.1%)	54 (51.9%)		57 (52.3%)	23 (47.9%)	
n.r.	9 (17.0%)	13 (12.5%)		13 (11.9%)	9 (18.8%)	
Intraoperative rupture	14 (26.4%)	27 (26.0%)	0.951	30 (27.5%)	11 (22.9%)	0.545
Treatment-related cerebral infarction	10 (18.9%)	24 (23.1%)	0.545	23 (21.1%)	11 (22.9%)	0.835

**Table 4:** Clinical and angiographic outcome. *mRS* modified Rankin scale

Variable	Standard working hours (*N* = 53)	On-call duty (*N* = 104)	*p*	Daytime (*N* = 109)	Nighttime (N = 48)	*p*
Vasospasm	31 (58.5%)	59 (56.7%)	0.833	66 (60.6%)	24 (50.0%)	0.218
Overall cerebral infarction	26 (49.1%)	45 (43.3%)	0.491	48 (44.0%)	23 (47.9%)	0.653
In-hospital mortality	9 (17.0%)	12 (11.5%)	0.343	14 (12.8%)	7 (14.6%)	0.768
Unfavorable outcome (mRS 3-6)						
At discharge	41 (77.4%)	65 (62.5%)	0.060	68 (62.4%)	38 (79.2%)	0.039
At 6-month follow-up	31 (58.5%)	49 (47.1%)	0.178	52 (47.7%)	28 (58.3%)	0.220
Aneurysm occlusion			0.689			0.933
Complete occlusion	22 (75.9%)	43 (71.7%)		46 (74.2%)	19 (70.4%)	
Neck remnant	6 (20.7%)	12 (20.0%)		12 (19.4%)	6 (22.2%)	
Aneurysm remnant	1 (3.4%)	5 (8.3%)		4 (6.5%)	2 (7.4%)	

### Operation start daytime vs. nighttime

Next, the patient cohort was stratified based on time of surgery. Microsurgical clipping was performed during daytime in 109 patients (69.4%) and during nighttime in 48 (30.6%). Among 83 patients admitted before 18:00, 26 (31.3%) were operated at night. Out of 74 patients admitted after 18:00, 22 (29.7%) underwent microsurgical clipping at the same night. In the other patients, surgery was delayed to the next day. There were no significant differences regarding baseline patient and aneurysm characteristics between both groups, as shown in Table 2. For surgeries performed during nighttime, the mean admission-to-surgery time (5.0 ± 3.9 h) was significantly shorter than that for surgeries during daytime (11.1 ± 6.9 h, *p* < 0.001), while the mean operation time (*p* = 0.337) showed no significant difference (Table 2). During nighttime, ICG-VAG was significantly less frequently used (75.0%) than during the daytime (89.9%, *p* = 0.015), while there was no significant difference in micro-Doppler usage (*p* = 0.203). Also, the frequency of temporary parent artery clipping (*p* = 0.966), intraoperative rupture (*p* = 0.545) and treatment-related cerebral infarction (*p* = 0.835) were comparable between both groups (Table 3). Vasospasm (*p* = 0.218) and overall cerebral infarction (*p* = 0.653) occurred in a similar portion of patients in both groups (Table 3). Infarction rates were slightly higher in patients treated without ICG-VAG (33.3%) compared to those treated with ICG-VAG (19.4%), however, this difference did not reach statistical significance (*p* = 0.434). Favorable outcome was significantly more often achieved after clipping during daytime (37.6%) compared to nighttime (21.8%, *p* = 0.039), while in-hospital mortality rates were comparable between the groups (*p* = 0.768). The difference in functional outcome was mitigated at 6-month follow-up (52.3% vs. 41.7%, *p* = 0.220). Complete and adequate occlusion rates at angiographic follow-up were similar in both groups (*p* = 0.689).

### Risk factors for unfavorable outcome at discharge

As surgery during nighttime was associated with a worse functional outcome at discharge, the impact of baseline patient characteristics, aneurysm characteristics and procedural specifics on functional outcome was further evaluated. In the univariate analysis—besides surgery during nighttime (*p* = 0.039)—higher patient age (< 0.001), WFNS 4+5 (*p* < 0.001), Fisher 4 (*p* < 0.001), longer admission-to-surgery time (*p* = 0.001), and overall cerebral infarction (*p* < 0.001) were predictive for unfavorable outcome. In the multivariate analysis, patient age (odds ratio [OR]: 1.05, 95%confidence interval [CI]: 1.01–1.09, *p* = 0.007), WFNS 4+5 (OR: 3.7, 95%CI: 1.2–11.7, *p* = 0.025), Fisher 4 hemorrhage (OR: 4.0, 95%CI: 1.6–10.1, *p* = 0.004), and overall cerebral infarction (OR: 3.5, 95%CI: 1.7–7.3, *p* = 0.001) were independently associated with unfavorable outcome. Aneurysm treatment during nighttime was not independently associated with poor outcome (OR: 2.1, 95%CI: 0.7–6.2, *p* = 0.169).

## Discussion

In the current study, on-call duty admission was not associated with increased morbidity or mortality. However, microsurgical clipping at nighttime carried higher odds of unfavorable outcome at discharge, representing a potential “weekend effect.” However, cerebral infarction rates were independent of the time of treatment and the difference in patient outcome was mitigated at mid-term follow-up. Furthermore, nighttime surgery was not independently associated with patient outcome after multivariable adjustment. To the best of our knowledge, this is the first clinical study that investigates the impact of a potential “weekend effect” on aSAH patients undergoing microsurgical clipping.

The weekend effect was first described in the benchmark study by Bell et al. in 2001 [3]. The authors performed a population-based study and revealed a higher mortality rate for 23 of 100 investigated nontraumatic causes of death among patients admitted on weekends [3]. Since then, the weekend effect has been evaluated for various traumatic and non-traumatic diseases [5, 6, 21, 23, 26, 34, 36]. Recently, Pauls et al. performed a meta-analysis of 97 studies with various types of emergency admissions, confirming increased odds of mortality (OR: 1.19, 95%CI: 1.14-1.23) for weekend versus weekday admission [32]. Several potential reasons for the weekend effect were discussed: During weekends, there is generally a shortage of both physicians and nurses, which is potentially associated with an increased individual workload. In this context, there is a decreased availability and performance of hospital services, such as imaging and interventional procedures [3]. Moreover, some authors proposed that physicians on duty might be less experienced with the management of a specific emergency case leading to suboptimal patient care [3, 32]. For patients with myocardial infarction, Kostis et al. demonstrated that patients admitted during on-call duty were less likely to receive invasive procedures in a timely manner [23].

Regarding aSAH, a potential weekend effect is possible given that the management of patients with an acute SAH require meticulous care, highly trained human resources, considerable technological resources and timely invasive procedures. In the available literature, however, there is conflicting evidence regarding this matter. In a population based study, Johnson et al. reported higher odds for mortality among SAH patients presenting at the weekend (OR: 1.07, 95%CI: 1.02-1.12) [22]. Likewise, Mikhail et al. observed higher mortality rates in SAH patients with a poor neurological grade (OR 6.59, 95% CI 1.62–26.88) [27]. In contrast, Pandey et al. and Crowley et al. found no association between weekend admission and mortality in population-based studies [8, 31].

The authors’ institution follows a “coil-first” policy; hence, microsurgical clipping is proportionally more often performed in patients with space-occupying intracranial hemorrhage and brain edema. In the multivariate analysis, a poor WFNS grade and a high Fisher grade were independently associated with unfavorable functional outcome. A good functional outcome at 6-month follow-up was achieved by 49%. For comparison, in the international subarachnoid aneurysm trial (ISAT), a good neurological outcome was reported for 69% in the clipping cohort [28]. However, the ISAT contained a preselected subset of patients with predominantly low-grade SAH. Interestingly, in the present study, on-call duty admission of aSAH patients was by trend associated with a better functional outcome at discharge, however, in-hospital mortality rates were similar and the difference in outcome was mitigated at midterm follow-up. After adjustment for confounding variables, on-call duty admission was not independently associated with morbidity. Moreover, there was no significant delay of start of surgery during on-call duty. The following considerations contradict a potential weekend effect in aSAH patients: Owing to a significant risk of aneurysm rebleeding and associated morbidity with each additional day of treatment delay, microsurgical clipping was performed within 24 h after admission both on weekdays and on weekends, which represents a key concept in SAH management [7]. Moreover, SAH patients are generally treated at specialized neurovascular units with standardized protocols. Upon admission, the patients are seen according to the same protocol regardless of day and time of the week they present, which includes immediate admission to a neurointensive care unit and evaluation by an interdisciplinary team of neurosurgeons, neurointerventionalists, and ICU physicians. For these reasons, the care of patients with SAH has become standardized and the management is familiar to the health care staff. The idea that standardized treatment protocols can mitigate the weekend effect has been demonstrated for ischemic stroke patients: McKinney et al. observed a weekend effect on ischemic stroke among non-stroke centers, however, comprehensive stroke centers that follow standardized treatment protocols were not affected [25]. These findings support the concept that standard operation procedures improve patient outcome, in particular at weekends with potentially less experienced health care workers being on duty.

We further hypothesized that clipping during nighttime would be associated with a worse surgical performance, which could result in a poor outcome. Due to the human circadian rhythm, cognitive performance varies throughout the day and usually reaches its lowest point at night, possibly yielding reduced overall quality of patient management [29]. Moreover, technical and personnel resources are particularly restricted at night. Concerning neurosurgical procedures, Desai et al. reported an increased morbidity and mortality rate among pediatric neurosurgical emergencies admitted during out-of-office hours [10]. Hirose et al. found that in-hospital mortality of emergency trauma patients was significantly higher during nighttime, however, there was no difference regarding weekdays and weekends [19]. In contrast, Rumalla et al. found no association between weekend admission and mortality among traumatic subdural hematoma patients [35].

Regarding aneurysm clipping, treatment-associated morbidity is mainly related to cerebral infarction, which can occur between 0.9 and 45.3% after clipping [4, 24, 38, 40]. The reasons for cerebral infarction include lengthy temporary clipping of the parent artery, occlusion of perforating arteries by improper clip placement and excessive brain retraction [2, 30, 33, 37]. In our series, treatment-related infarction occurred in 21.7% and was comparable between the daytime and the nighttime group. In this context, there were no differences regarding temporary parent occlusion and vasospasm. Also, operation time was comparable between both groups, which might be considered an argument against surgeon’s fatigue at nighttime. These considerations are further supported by studies which demonstrated that sleep deprivation has no impact on surgeon performance [39, 41]. However, ICG-VAG was less often used at nighttime, which may be explained by personal preferences and limited resources (e.g. unexperienced staff). Infarction rates were slightly higher in patients treated without ICG-VAG; however, this difference was not statistically significant.

Although treatment-related complications were similar between daytime and nighttime surgery, patients treated at nighttime had a higher rate of unfavorable outcome at discharge. However, this difference did not remain significant after multivariable adjustment. In conclusion, we could not demonstrate a statistically significant effect of on-call duty admission and microsurgical clipping at nighttime on in-hospital mortality and mid-term functional outcome. Although, we observed worse short-term functional outcome after nighttime surgery, there were no differences in complications, vasospasm and infarction rates, making a considerable weekend effect appear unlikely. Although we could not prove a “weekend effect” for the analyzed subset of aneurysms, there might be a “weekend effect” in other institutions that follow a different treatment regimen or in specific subsets of patients with diverging baseline characteristics. Further studies will be required to draw a definite conclusion on this subject.

### Limitations

The study is limited by its retrospective, single center design. The number of included patients was only moderate, and it would be possible that some results could achieve statistical significance with increasing statistical power. We report the outcome of patients from our neurovascular center, which underwent a standardized management protocol. Therefore the results may not apply to other institutions with different treatment regimen. Moreover, we did not analyze aneurysm re-rupture, which needs to be considered when deciding to delay aneurysm embolization.

## Conclusions

In this study, clipping during nighttime was associated with worse patient outcome at discharge. However, this effect did not remain significant after multivariable adjustment. Moreover, in-hospital mortality rates and procedural complications were not different between daytime and nighttime. In our neurovascular center, admission outside of regular working hours did not affect patient outcome, which is most probably due to the following of standardized treatment protocols.
